# Planar Cell Polarity: A Bridge Too Far?

**DOI:** 10.1016/j.cub.2008.09.002

**Published:** 2008-10-28

**Authors:** Peter A. Lawrence, Gary Struhl, José Casal

**Affiliations:** 1 Department of Zoology, University of Cambridge, Downing Street, Cambridge CB2 3EJ, UK; 2 MRC Laboratory of Molecular Biology, Hills Road, Cambridge CB2 0QH, UK; 3 Howard Hughes Medical Institute, Columbia University College of Physicians and Surgeons, 701 West 168th Street, New York, New York 10032, USA

## Abstract

The mechanisms of planar cell polarity are being revealed by genetic analysis. Recent studies have provided new insights into interactions between three proteins involved in planar cell polarity: Flamingo, Frizzled and Van Gogh.

## Main Text

We now understand much of how cells know where they are in an embryo, but little of how they know their orientation, anterior from posterior, distal from proximal. Yet we believe that many, perhaps all, epithelial cells are polarised in the plane of the sheet — that they exhibit planar cell polarity, and that this polarity is vital. Planar cell polarity is not used primarily to *make* structures but more to *orient* them, making its study conceptually difficult. But, genetics is the right approach and *Drosophila* has proved the model of choice — particularly as the genes identified in the fly are conserved in other animals, including vertebrates [1–3]. In the 60s it was argued that pervasive gradients are set up in the main axes of the body; it was suggested that the slope of a gradient could specify the polarity of cells [4,5]. This viewpoint is still very much alive and these gradients are now being identified with the help of genetics. There is now a resurgence of interest in the mechanisms of planar cell polarity: three new papers [6–8] (one in this issue of *Current Biology* [7]) report the use of both genetics and molecular techniques to get to one of the two hearts of the matter.

*Drosophila* cells make oriented structures; examples are hairs and bristles on the wing and abdomen. In the 80s, pioneers such as Adler and Gubb found genes whose mutants altered these polarities [9,10]. Early on *frizzled* (*fz*) was identified; and, significantly, it was found that clones of *fz^−^* cells repolarised neighbouring wild-type cells so that they point their hairs towards cells with lower Fz activity [9,10]. It helps to think of the *fz^−^* cells as *sending* and the wild-type cells as *receiving* polarising information [11]. Many different genetic mosaics can be made in *Drosophila* and, for example, each gene can be tested to see if it is needed in the sending, in the receiving cells or in both. This repolarisation assay has proved an incisive aid in the analysis of planar cell polarity.

The first working models used a small group of genes: *prickle* (*pk*), *fz*, *Van Gogh* (*Vang*) (also called *strabismus*, *stbm*) and *dishevelled* (*dsh*). In the 90s it was found that, just before polarised structures are formed, some of these proteins become localised to one or other ends of the cell [12]. It was suggested that some small initial bias (unknown) is amplified by interactions and feedback between these four proteins to polarise each cell; propagation from cell to cell would be driven by interactions across the intercellular space [13]. This model was simulated in a powerful computer [14] and became popular; however, complex computers are no match for simple experiments and the model looked feeble when it was found (in repolarisation assays) that *pk* and *dsh* are dispensable in *both* sending and receiving cells and so, for this central process, could be ignored [11,15–17]. The model suffered further blows when we found that a cell completely lacking *fz* could be repolarised [11] and that protein localisation itself appeared to be dispensible for repolarisation [11,16].

**Figure 1:**
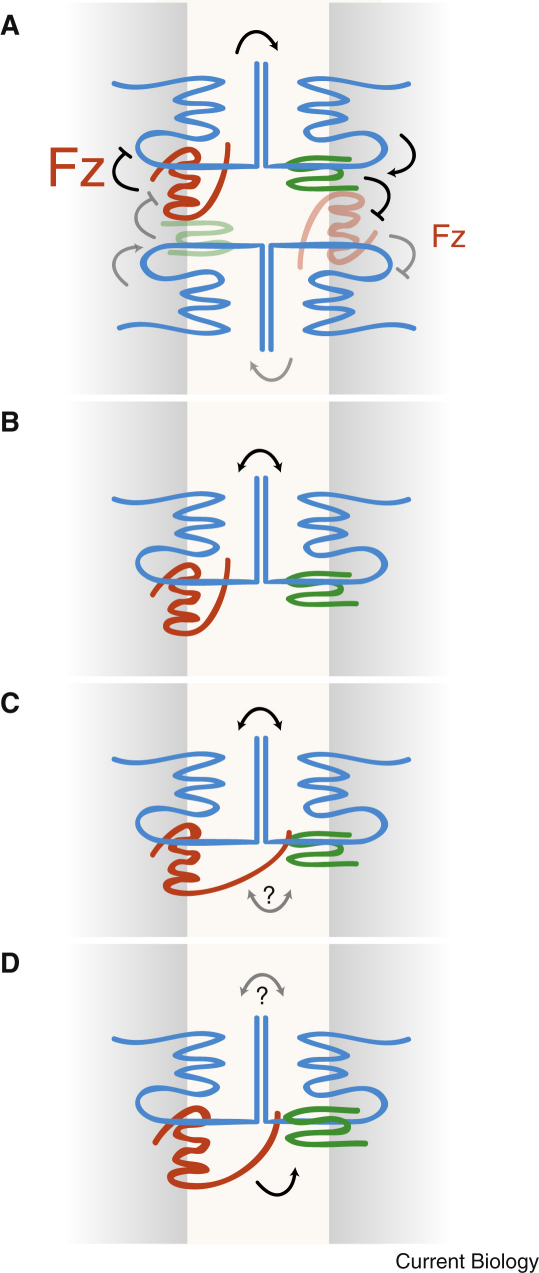
Possible intercellular and intracellular relations between Fmi (blue), Fz (red) and Vang (green), according to different authors. Two membranes from neighbouring cells are shown. If a direct physical interaction is suggested, the molecules are shown in contact. In (A), the size of lettering refers to the original gradient of Fz activity, with the strength of colour of the molecules indicating the subsequent localisation of the proteins. The arrows show the direction of the flow of information: black is strong, grey is weak signalling. (A) Lawrence *et al.*[11]; (B) Chen *et al.*[6]; (C) Strutt and Strutt [7]; (D) Wu and Mlodzik [8].

*Flamingo* (*fmi*, also known as *starry night* or *stan*), was largely left out of these models. In our assays, however, it was the only gene needed in *both* sending and receiving cells and, because its protein product is able to form homodimers from one cell to the next [18], we placed it at the centre of a new model [11]. In our model, the Fmi homodimers act as intercellular bridges. We suggested that, using Fmi to compare its neighbours, each cell points its hair towards the neighbour with the lowest level of Fz activity, and that there is an intercellular feedback via Fmi, which brings the level of Fz activity in one cell towards an average of its neighbours. We argued that Fmi–Fmi homodimers act asymmetrically to convey the level of Fz activity in the sending cell to Vang in the receiving cell. Because information is actually going in both directions — in the wild-type, each cell will both send and receive — it follows Fmi can act in two ways in the same cell depending on whether it sends (with Fz) or receives (with Vang) (Figure 1). A more detailed version of this model was elaborated subsequently [2] and another similar one simulated *in silico* [19].

Chen *et al.* [6] recently reported the results of similar experiments to ours [11] but, instead of monitoring hairs, they mostly looked at localisation of the proteins, a concordant indicator of polarity. They reached the same conclusions as we did [11], namely that Fmi is needed in both sending and receiving cells, placing Fmi centrally in planar cell polarity. They also shifted their attention from Pk and Dsh, conceding (though not stating) that their earlier models have been superseded. So we now have a model in which homodimers of Fmi make intercellular bridges, and, as the three new papers [6–8] make clear, a new question of whether these bridges are conduits for polarising information, or are more passive, for example helping Fz and Vang to contact each other as ligand and receptor.

Chen *et al.* [6] believe direct contact is implausible because of the wide gulf between the cells. If they are right, Fz might be able to mediate planar cell polarity without the cysteine-rich domain (CRD), part of the protein's amino-terminal ectodomain, and Chen *et al.* [6] offer some evidence for this. But their evidence conflicts with previous findings that the Fz ectodomain is essential for planar cell polarity [20]. Also, in contrast to Chen *et al.* [6], and with stronger evidence, Wu and Mlodzik [8] find once again that the Fz CRD is essential. Extending the disagreement between the two papers, Wu and Mlodzik [8] not only find the interaction of Fz and Vang quite plausible, they actually find direct binding using pulldowns and binding assays in tissue culture cells — but they do not determine whether the binding is in *trans* (from cell to cell, as they assume) or in *cis* (which would fit with our evidence that Vang acts in *cis* to regulate Fz [11]). The data reported by Strutt and Strutt [7] agree with Wu and Mlodzik [8], providing independent evidence from tissue culture experiments that Vang and Fz can bind in *trans*, a binding that is enhanced in the presence of Fmi.

In spite of the possible significance of direct intercellular binding of Vang and Fz, we know that Fmi is essential for propagation of planar cell polarity information — so how do the three proteins relate? Chen *et al.* [6] describe evidence from immunoprecipitation that Fz binds to Fmi, and with this Strutt and Strutt [7] agree. But none of the groups shows biochemical evidence of binding between Vang and Fmi. Also, using overexpressed Fmi *in vivo*, Strutt and Strutt [7] report that Fmi needs both Vang and Fz if it is to stabilise properly in the cell membrane and that it prefers to bind to Fz rather than to Vang. These mixed observations tie Vang, Fz and Fmi closely together but they do not tell us clearly how the information about Fz activity is exchanged, nor what are the structural or functional relationships between the three proteins.

Chen *et al.* [6] ask whether Fmi acts actively (as we previously proposed [11]) or passively (as now proposed by Wu and Mlodzik [8]). They claim to answer by showing that *Vang^−^ fz^−^* sending cells do repolarise receiving cells when they strongly overexpress *Fmi* and argue that this means that Fmi can act instructively. However, this argument is undermined by the conflicting and positive finding of Wu and Mlodzik [8] that *Vang^−^ fz^−^* sending cells repolarise receiving cells, even without overexpressing Fmi. The contradictory results and conclusions described in the three papers [6–8] show that we are not yet ready to answer this enigmatic question.
